# Upper limb neuropathy in computer operators? A clinical case study of 21 patients

**DOI:** 10.1186/1471-2474-5-26

**Published:** 2004-08-13

**Authors:** Jørgen Riis Jepsen

**Affiliations:** 1 Department of Occupational Medicine, Sydvestjysk Sygehus, Østergade 81-83, DK-6700 Esbjerg, Denmark

## Abstract

**Background:**

The character of upper limb disorder in computer operators remains obscure and their treatment and prevention have had limited success. Symptoms tend to be mostly perceived as relating to pathology in muscles, tendons or insertions. However, the conception of a neuropathic disorder would be supported by objective findings reflecting the common complaints of pain, subjective weakness, and numbness/tingling. By examining characteristics in terms of symptoms, signs, and course, this study aimed at forming a hypothesis concerning the nature and consequences of the disorder.

**Methods:**

I have studied a consecutive series of 21 heavily exposed and severely handicapped computer-aided designers. Their history was recorded and questionnaire information was collected, encompassing their status 1/2 – 1 1/2 years after the initial clinical contact. The physical examination included an assessment of the following items: Isometric strength in ten upper limb muscles; sensibility in five homonymously innervated territories; and the presence of abnormal tenderness along nerve trunks at 14 locations.

**Results:**

Rather uniform physical findings in all patients suggested a brachial plexus neuropathy combined with median and posterior interosseous neuropathy at elbow level. In spite of reduced symptoms at follow-up, the prognosis was serious in terms of work-status and persisting pain.

**Conclusions:**

This small-scale study of a clinical case series suggests the association of symptoms to focal neuropathy with specific locations. The inclusion of a detailed neurological examination would appear to be advantageous with upper limb symptoms in computer operators.

## Background

Upper limb pain and dysfunction are frequent complaints associated with computer work. However, the responsible pathology and the pathophysiological mechanisms are insufficiently understood. In addition, there is no consensus with regard to physical findings that may reflect symptoms.

The involvement of the nerves in "non-specific" upper limb disorder, e.g. in computer operators, is suggested by various observations: The demonstration of an elevated threshold to vibratory stimulation [1-3]; abnormal upper limb tension tests [4,5]; reduced nerve mobility [6,7]; abnormal nerve tenderness (mechanical allodynia) [8]; changed axonal flare reaction [9]; allodynic response to supra-threshold vibration [2]; reduced muscle strength [10,11] and sympathetic reflexes [12]; and thermographic changes [13]. Still, clinical practice and epidemiological studies tend to attribute upper limb symptoms in computer operators to a disorder in muscle, tendon, or insertion [14]. Focal neuropathy including carpal tunnel syndrome is infrequently reported [15,16].

Upper limb pain in computer operators shares the features of a neuropathic pain: Common analgesics tend to be ineffective. Pain may be evoked spontaneously or may appear to constitute an abnormal response to stimuli with frequent occurrence of allodynia. In addition, there are often non-painful abnormal spontaneous or evoked sensory phenomena such as numbness/tingling. The common experience of weakness which may further deteriorate on use would also be compatible with an upper limb nerve affliction.

A precise and accurate diagnosis is crucial for effective management and rehabilitation, and also for epidemiological studies concerning causation. In order to get a better understanding of the pathophysiological mechanisms, the injured tissue should be precisely located. This might not necessarily be where symptoms predominate.

I have aimed at studying a clinical series of computer operators with upper limb complaints and dysfunction in terms of

• exposure characteristics;

• symptoms and past treatment;

• physical findings which may reflect an affliction of the peripheral nerves;

• prognosis with regard to symptoms and work-status.

## Methods

### Patients

This study comprises a consecutive series of 21 computer-aided designers with pain and functional limitations in the dominant upper limb. All patients were referred to a department of occupational medicine for diagnostic and aetiological assessment and management. Three patients were males of median age 27 years (range 25–41) and 19 were females of median age 35 years (range 25–55).

### Clinical examination and interpretation

#### Interview

Patients were interviewed about the character, distribution, initial presentation and development of their symptoms. Special attention was given to the presence of upper limb pain, subjective weakness and numbness/tingling, and to other symptoms included in a standard protocol for work-related upper limb disorders [17].

#### Physical examination

A subsequent physical examination included extracts of diagnostic criteria for selected clinical disorders (tension neck syndrome, cervical syndrome, supra-and infraspinous tendinitis, bicipital tendinitis, frozen shoulder, acromioclavicular arthrosis, epicondylitis, tenosynovitis, and wrist and forearm peritendinitis) [17].

**Figure 1:**
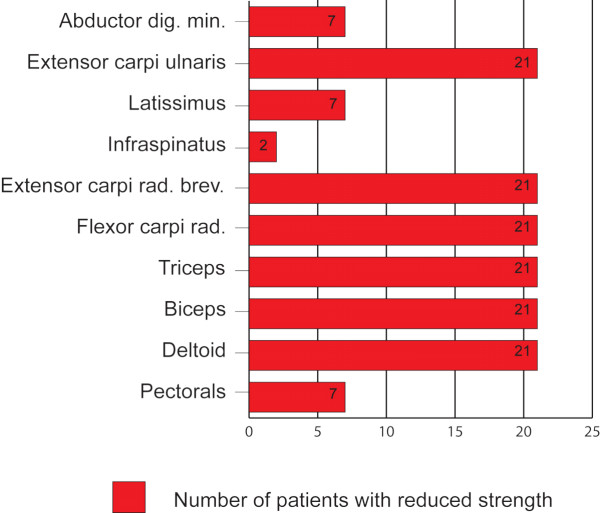
Strength reductions in 21 dominant limbs.

**Figure 2:**
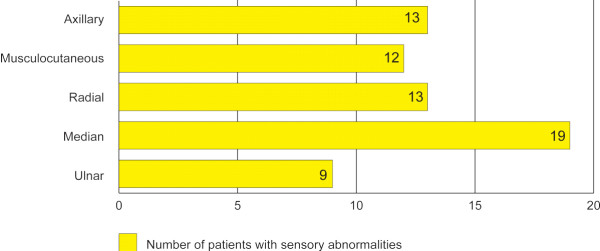
Sensory abnormalities (pain, touch, and/or vibratory sense) in 21 dominant limbs.

Upper limb nerve afflictions were defined from an additional neurological examination consisting of the following components:

• Manual assessment of the isometric strength in a selection of ten upper limb muscles (Figure 1). Any reduction of strength was registered as weakness [18,19]. Patients were encouraged to provide maximal muscle effort on both sides for each muscle tested, despite any potential discomfort.

• The sensibility (algesia by pinprick, aestesia by moving touch [19]) was assessed in five homonymous innervation territories (Figure 2):

• The axillary nerve (the deltoid area);

• The musculocutaneous nerve (the dorsal forearm);

• The radial nerve (the first dorsal web);

• The median nerve (the tip of the second finger);

• The ulnar nerves (the tip of the fifth finger).

*• The perception of vibration (tuning fork 256 Hz*[20]) *was additionally estimated in the ulnar and median territories (tips of second and fifth fingers).*

Any sensory deviation from normal was registered as abnormal.

• Assessment of tenderness with slight pressure at 14 locations along the course of nerves [8]. Any mechanical allodynia was registered as abnormal:

• The brachial plexus (scalene triangle, passage behind the pectoralis minor muscle);

• The suprascapular nerve (suprascapular notch);

• The axillary nerve (quadrilateral space);

• The musculocutaneous nerve (passage through the coracobrachial muscle);

• The median nerve (just proximal to the elbow, at the passage between the two heads of the pronator teres muscle, at the passage below the arcade of the common superficial flexor muscle, and at the carpal tunnel);

• The radial nerve (triceps and brachioradial arcades);

• The posterior interosseous nerve at the arcade of Frohse (supinator tunnel);

• The ulnar nerve (sulcus of the ulnar nerve and Guyon's canal in the hypothenar).

Assessments in patients with unilateral disorder were based on comparison to contra-lateral findings defined as normal. In patients with bilateral disorder, test-results were related to other findings in the same limb assumed to be normal, e.g., strength in adjacent muscles or sensibility in adjacent innervation territories [21].

The definition and location of a nerve affliction ("neuropathy") was based on a traditional approach with a focus on the topography and innervation patterns of the upper limb nerves. Special consideration was given to the presence of normal strength in certain muscles and of reduced strength in others [18,19], and to localized mechanical allodynia at the appropriate location(s) along the nerve trunk [8].

I have operated with two sets of criteria for the definition of focal neuropathy assuming the second criterion to be more convincing:

• *Criterion 1: *The presence of a pattern of muscle-weakness suggesting a focal neuropathy at a defined location, at which mechanical allodynia with slight pressure at the nerve is present.

• *Criterion 2: *Criterion 1 plus sensory deviations from normal in one or several sensory territories located peripherally to focal neuropathy.

In addition, double crush [22] at the appropriate location(s) was arbitrarily defined when strength reductions and/or mechanical allodynia were either equivalent or more prominent distally. The double crush theory refers to the phenomenon that a focal neuropathy increases the vulnerability of the nerve as a whole, resulting in a tendency of focal neuropathy to occur at several locations along the course of a nerve.

### Location of neuropathy

*Brachial plexus neuropathy at chord level *was defined with reduced strength in the deltoid, biceps, and radial flexor of the wrist muscles, when weaknesses were accompanied by brachial plexus tenderness at its passage behind the pectoral muscle. Depending on the extent of brachial plexus involvement, additional muscles may be weak and mechanical allodynia may extend in the proximal or medial direction.

*Median neuropathy at elbow level *was defined with reduced strength in the radial flexor of the wrist muscle along with mechanical allodynia involving the median nerve at elbow level (at the passage proximal to the elbow, between the two heads of the pronator teres muscle, and/or at the arcade of the superficial flexor of digits muscle). With an isolated median neuropathy, the deltoid, biceps, and ulnar extensor of the wrist muscles must be intact.

Double crush involving the brachial plexus and the median nerve was defined in the following situations:

• Strength in the radial flexor of the wrist muscle was reduced as much as / more than it was in the deltoid or biceps muscles.

• Mechanical allodynia was either the same or more conspicuous at the median nerve at elbow level than it was at plexus level.

*Posterior interosseous neuropathy *was defined with reduced strength in the ulnar extensor of the wrist muscle along with tenderness at the nerve-passage below the arcade of Frohse in the dorsal proximal forearm. With an isolated posterior interosseous neuropathy, the deltoid, biceps, short radial extensor of the wrist, and radial flexor of the wrist muscles must be intact.

Double crush involving the brachial plexus and the posterior interosseous nerve was defined in the following situations:

• Strength in the ulnar extensor of the wrist muscle was reduced as much as / more than in the deltoid, biceps, or radial flexor of wrist muscles.

• Mechanical allodynia was either the same or more conspicuous at the arcade of Frohse than it was at plexus level.

*Other potential focal neuropathy *was defined according to similar criteria, e.g., an isolated carpal tunnel syndrome would require reduced strength in the short abductor of the wrist muscle but preserved strength in the radial flexor of the wrist muscle. An isolated ulnar neuropathy at elbow or wrist level would require reduced strength in the abductor of the fifth digit and intact proximal muscles. In addition, mechanical allodynia should be present at the appropriate locations along nerve trunks.

### Management

Patients were recommended to freely move and use the symptomatic upper limb within the limits of immediate and subsequent pain aggravation. All patients were offered physiotherapy based on the concept of adverse neural tension [23,24] and encouraged to return to computer work after optimizing the work station ergonomics and work organization. Patients unable to return to work were advised concerning rehabilitation: Maximizing variation during future work; keeping the upper limbs close to the body; and avoiding repetition and static postures.

### Questionnaire

1/2 – 1 1/2 years after the initial examination the patients responded to a questionnaire: The exposure characteristics; symptoms (pain, weakness/fatiguability, numbness/tingling); past treatment; pain intensity at the first encounter and at follow-up to be quantified on a VAS-scale from 0 (no pain) to 10 (extreme pain); and the present status with regard to functional limitations and work.

### Statistics

The change of the level of reported pain between the first consultation at the department and at follow-up was assessed by Friedman's test.

## Results

### Exposure characteristics

All 21 patients returned the questionnaire. The mean duration of work with computer-aided design was 95 months (16–260 months). The self-reported daily mean of time spent with computer work constituted 81% (50–100%) of the total working time. 86% of the respondents reported aggravating factors during the months prior to the onset of symptoms, including high work intensity, overwork or other work conditions causing an unusual strain.

### Symptoms and past treatment

Pain in the dominant upper limb was common to all patients. It had a mean duration of 24 months (1–60 months) and was the main symptom in 13 patients. All but one patient had a subjective feeling of weakness/fatiguability. Five patients reported this to be the most disturbing symptom. 19 patients experienced numbness/tingling which constituted the main symptom in three of them. Five patients had bilateral symptoms.

All patients had received treatment prior to admission: A limited and transitory effect of past physiotherapy was reported in four out of 17, of pain killers in one out of 10, and of local steroid injections in two out of three patients. For the remaining patients the past treatment had no effect.

### Physical examination

According to the defined criteria for work-related upper limb disorders [17], non-neuropathic disorders were not identified.

In all 21 patients reduced strength was demonstrated in the following muscles: Deltoid, biceps, triceps, and the radial flexor, short radial extensor and ulnar extensor of the wrist. In a smaller number of patients there were additional strength-reductions in the pectoral, infraspinatus, latissimus and abductor of the fifth digit muscles (Figure 1).

Sensory abnormalities were identified in 19 out of 21 patients. The median nerve territory was most frequently involved. However, most patients had additional sensory deviations in territories innervated by the radial, musculocutaneous, axillary, or ulnar nerves (Figure 2).

**Figure 3:**
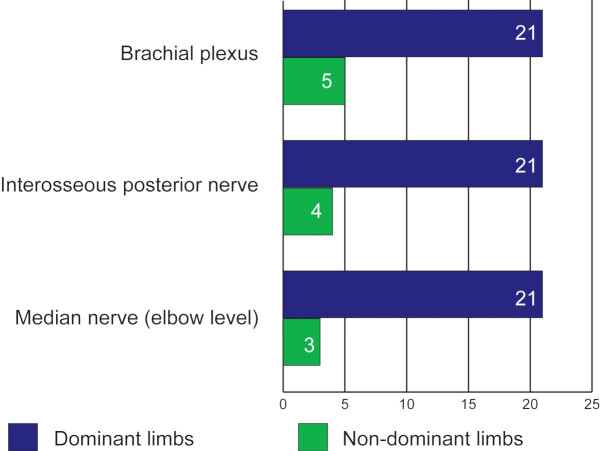
Mechanical allodynia with mild pressure at three locations along nerves in 21 dominant limbs compared to the contra-lateral limb.

In all patients, mechanical allodynia was present at the brachial plexus at chord level, i.e., on its passage behind the pectoral muscle. In two patients it was also present at trunk level, i.e., at the scalene triangle. Furthermore, mechanical allodynia was present in all patients at the posterior interosseous nerve at the arcade of Frohse, and at the median nerve at one or several locations around the elbow. No mechanical allodynia was observed at the suprascapular, axillary, musculocutaneous, ulnar or radial nerves, nor at the median nerve at the volar wrist (Figure 3).

Contra-lateral mechanical allodynia was present at the brachial plexus in five patients, and additionally at the posterior interosseous and median nerves in four and three of these patients, respectively (Figure 3). The five patients with contra-lateral mechanical allodynia had bilateral complaints.

**Figure 4:**
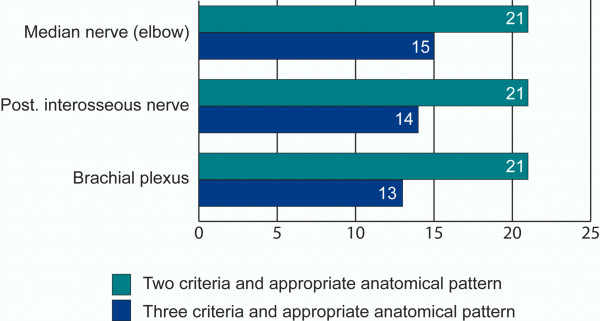
Definition according to the applied criteria of focal neuropathy in 21 dominant limbs.

According to the defined criteria, the patterns of physical findings in all 21 dominant limbs suggested the presence of a brachial plexus neuropathy in combination with a median and posterior interosseous neuropathy at elbow level (Figure 4). No other nerve entrapments were defined in this sample.

### Prognosis

At follow-up after 1/2 – 1 1/2 years, only two out of the 21 patients remained in computer work. Three were employed in other jobs while the majority was training for other jobs (eight patients) or unemployed (eight patients).

**Figure 5:**
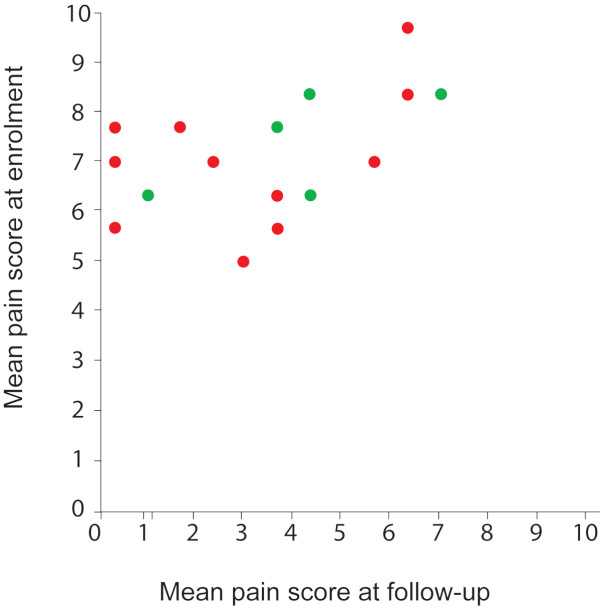
**Change of pain from enrolment until follow-up in 21 dominant limbs. **The mean pain scores when pain was worst and when pain was least is indicated for each patient at enrolment and at follow-up. Red dots represent one case. Green dots represent two cases.

Eleven patients reported a beneficial effect of the proposed physiotherapy, five experienced no effect, and five did not pursue the suggested treatment. On a group basis, the mean of pain when worst was reduced from 8.0 at enrolment to 6.1 at follow-up. The corresponding figures when pain was least were 4.3 and 2.7, respectably. This reduction was significant (χ^2 ^= 8.0 and 9.0, p < 0.005 and 0.003, respectively). However, the pain persisted on a disturbing level in the majority of patients (Figure 5). The severity of pain at follow-up was unrelated to the present occupational status and to the severity of pain at enrolment. Neither of the two parameters was related to sex, age, or the duration of exposure.

## Discussion

Working in computer-aided design involves an almost continuous operation of the pointing device for extended periods of time. Consequently, the dominant upper limb strain is more pronounced than in other computer work. Following this exposure, all the patients were severely handicapped in this very limb. In most patients, the unilateral disorder enabled the examiner to conveniently compare the outcomes of the physical examination in the dominant limb with the contra-lateral findings.

There is a general consensus that reduced muscle strength, sensory deviations from normal, and localized mechanical allodynia are related to afflicted peripheral nerves [19]. The relation to an underlying neurological change is indicated by the occurrence of these abnormalities in patterns, in accordance with anatomical facts.

The complaints of pain in all dominant upper limbs, and subjective complaints of weakness and numbness/tingling in most of them, were reflected by a rather uniform pattern of strength-reductions, mechanical allodynia, and sensory deviations from normal suggesting the involvement of the brachial plexus at chord level and of the posterior interosseous and median nerves at elbow level. In this sample, I found no indication of carpal tunnel syndrome, ulnar neuropathy at elbow or wrist level, radial neuropathy before division of the posterior interosseous nerve, nerve root compression, or any other neuropathic or non-neuropathic upper limb disorder.

Abnormalities were detected by tests which are included in the classical neurological examination. To enable the examiner to assess a single muscle at a time, the limb position during testing should aim at limiting disturbing interference from other muscles [21]. The mostly minor strength reductions demanded the quantification of strength-reductions to include Grade 4+ (Contraction against gravity and strong resistance [18]). The identification of such minor weakness demands simultaneous testing on the right and the left side. The absence of facial expressions, withdrawal, or complaints from the patients suggested that the voluntary contraction during strength testing was not influenced by simultaneous pain.

The physical examination in this clinical case study was not blinded concerning patient-related information, such as the presence and location of symptoms. However, in a recent validation of the physical tests applied we have found that blinded examiners could reliably assess the individual items (individual muscle strength [21], sensory qualities, mechanical allodynia), as well as the occurrence of findings in patterns in accordance with the course of nerves and the innervated tissue. Findings were also reflected by the presence of symptoms.

Clinical experiences have led to hypotheses suggesting upper limb symptoms in computer operators to be related to prolonged non-neutral and predominantly static positions including a flexion of the shoulder and a submaximally pronated forearm. This may result in a muscular imbalance from some muscles being successively shortened and their antagonists passively stretched and weakened [25]. Pain and functional limitations may result from limited available space for the nerves especially at locations close to joints or adjacent to bony prominences, fibrous bands or tunnels. This may cause tension, friction, and compression [24,25]. Reduced axoplasmatic flow at a proximal site may lessen the ability of nerves to withstand adverse forces at a more distal site (or the reverse), as described in the double crush phenomenon [22], and the mobility of the entire nerve may be impaired by such external affliction [6,24,26].

From the topography of the brachial plexus, the lateral chords would appear to be most at risk behind the pectoralis minor muscle. The muscles supplied from this part of the plexus (deltoid, biceps, radial flexor of wrist, triceps, short radial extensor and ulnar extensor of the wrist) were invariably involved. In a few limbs there was an additional involvement of the pectoral, small abductor of the fifth digit, latissimus dorsi, and infraspinatus muscles (Figure 1). This is concurrent with a medial or proximal extension of a brachial plexus affliction.

Caution should be exercised when drawing a comparison between the outcome of this study of patients, referred with a serious disorder, and studies of "healthy" computer operators in occupation. Still, it would be relevant to compare with upper limb findings in computer operators described by others. A study of 533 visual display terminal workers has suggested an array of upper limb disorders in 22%, dominated by tendon related conditions in 15% and probable nerve entrapment in 4% [15]. In a study of 632 newly hired computer operators, the one year incidence of neck and shoulder symptoms was 58% and of hand/arm symptoms 39%. Symptoms were explained by physical findings in the neck/shoulder region in 35% of participants ("somatic shoulder/neck syndrome" in 33%) and in the hands/arms in 21% (de Quervain's syndrome in 15%) [27]. In a recent major cross-sectional study of almost 7000 computer operators, 20% complained of moderate to severe pain. The physical examination, however, was only able to disclose a limited number of upper limb disorders, similar to what would be expected in the general population [28]. Self-reported numbness/tingling in 10.9% of the computer operators was attributed to carpal tunnel syndrome in a minority (numbness/tingling in the median nerve territory in 4.8%, and symptoms at night in 1.4%) and unexplained in the remaining subjects [16]. Based on the same material, nerve entrapment was only diagnosed in 12 subjects (supinator syndrome and pronator syndrome defined by localized palpation tenderness with withdrawal, and pain with provocative maneuvers). No new cases of nerve entrapment occurred during a one year follow-up [29].

However, the diagnoses depend on the choice and validity of the clinical tests employed and on the diagnostic criteria applied. The "somatic shoulder/neck syndrome" [27] is characterized by nonspecific signs and may well be a neuropathic condition. Discomfort with the Finkelstein maneuver [15,27] is not specific for de Quervain's syndrome [30]. If not associated with first dorsal compartment tenderness and swelling, this diagnosis would seem to be unjustified. The common occurrence of de Quervain's syndrome in computer operators who hardly move their thumb would seem unlikely.

My findings are more in accordance with those of Pascarelli, who studied 485 upper limb patients out of which 70% were computer operators. A detailed and comprehensive physical examination demonstrated protracted shoulders in 78% and head forward position in 71%. This was also frequent in my study-patients but not systematically registered. A neurogenic thoracic outlet syndrome in 70% was suggested by tests stressing the brachial plexus and by the demonstration of mechanical allodynia [31]. In a former study of 53 computer operators with severe upper limb disorders Pascarelli has found a high prevalence of reduced muscle strength and impaired passive wrist deviation associated with an increase in forearm pain. These findings were attributed to myofascial shortening and found to be useful clinical indicators of injury [32].

## Conclusions

The limited success of the prevention and management of computer-related upper limb disorders demands new approaches to practice and research in the field. The inclusion in future studies of the presented systematic examination of the upper limb nerves may provide additional diagnostic information. This may lead to future improvement of the prevention and management of computer-related upper limb disorders.

## Competing interests

None declared.

## Authors' contributions

JRJ designed the study and conducted all the clinical examinations, and wrote the manuscript.

## Pre-publication history

The pre-publication history for this paper can be accessed here:
